# Sturge–Weber syndrome with intracerebral hemorrhage: a case report

**DOI:** 10.1186/s40064-016-3439-z

**Published:** 2016-10-07

**Authors:** Masashi Chonan, Yasuhiro Suzuki, Shinya Haryu, Shoji Mashiyama, Teiji Tominaga

**Affiliations:** 1 Department of Neurosurgery, Iwaki Kyoritsu Hospital, 16 Kusehara, Uchigo Mimaya-machi, Iwaki, Fukushima 973-8555 Japan; 2 Department of Neurosurgery, Tohoku University Graduate School of Medicine, 1-1 Seiryo-machi, Aoba-ku, Sendai, 980-8575 Japan

**Keywords:** Sturge–Weber syndrome, Intracerebral hemorrhage, Sinus thrombosis

## Abstract

**Introduction:**

Sturge–Weber syndrome (SWS) is a rare congenital disease that affects the brain, skin, and eyes, and is a sporadically occurring neurocutaneous syndrome that affects intracerebral veins, which is associated with venous thrombosis. However, intracranial hemorrhage in patients with SWS is rare. We herein report a rare case of SWS with intracerebral hemorrhage derived from sinus thrombosis.

**Case description:**

A 62-year-old man suddenly fell into a coma and was admitted to our hospital. His neurological status was assessed as GCS 6 (E1V1M4) with right-sided hemiparesis. At birth, he had a right-sided facial port-wine stain typical of SWS that involved the ophthalmic division of the trigeminal nerve. Laboratory findings showed that he was dehydrated, and his serum D-dimer concentration was increased. Computed tomography revealed left thalamic hemorrhage with acute hydrocephalus and cortical calcification in the right occipital lobe. Magnetic resonance imaging displayed a vascular malformation of the right cerebral hemisphere consistent with SWS. Magnetic resonance venography showed steno-occlusion of the superior sagittal sinus, straight sinus, and left internal cerebral vein (ICV). Emergency ventricular drainage was performed. Seven days after surgery, his consciousness improved to GCS 14 (E4V4M6). Rehydration therapy was performed to prevent sinus thrombosis.

**Discussion and Evaluation:**

His postoperative course was uneventful. Sudden congestion of the left ICV may have caused left thalamic hemorrhage.

**Conclusions:**

SWS with major sinus occlusion needs to be diagnosed with utmost caution in order to allow for preoperative neurological and radiological assessments.

## Background

Sturge–Weber syndrome (SWS) is a rare sporadic neurocutaneous syndrome that appears to be caused by a somatic mutation in *GNAQ*, and affects early fetal vascular development (Shirley et al. 2013). SWS affects male and female newborns at an incidence of approximately 1 in 20,000–50,000 births. This syndrome is defined by a unilateral or bilateral facial port-wine stain that involves the ophthalmic division of the trigeminal nerve with vascular abnormalities of the brain including ipsi-lateral leptomeningeal angiomatosis and angioma in the ipsilateral eye. Patients commonly have ophthalmological and neurological clinical features including glaucoma, visual field deficits, seizures, stroke-like episodes, migraine-like headaches, and cognitive delays. Most cases of SWS are not life-threatening.

Leptomeningeal angiomatosis results in a vascular steal affecting the subjacent cortex and white matter producing localized ischemia. The deep venous occlusion of venous abnormalities in SWS is rare (Slasky et al. 2006). Only a few cases of SWS with intracranial hemorrhage have been reported to date (Table 1). We herein present a rare case of SWS with intracerebral hemorrhage derived from sinus thrombosis.

**Table 1:** Reported cases of Sturge–Weber syndrome with intracranial hemorrhage

Case no.	Author year	Age	Sex	Premorbid neurological status	Location of hemorrhage	Presumed etiology	Signs and symptoms	Outcome
1	Anderson and Duncan (1974)	32 years	Female	Normal	Subarachnoid hemorrhage	Angiomatous malformation in the *left* basal ganglia and thalamic regions	Headache, Increased deep tendon reflexes, nuchal rigidity	Free of seizures and headaches
2	Pozzati et al. (1983)	9 years	Male	Epilepsy	*Left* parieto-occipital intracerebral hemorrhage	*Left* posterior cerebral artery aneurysm	Headache, hemiparesis, homonymous hemianopsia	Free of seizures, stable hemiparesis
3	Dolkart and Bhat (1995)	24 years	Female	Epilepsy	*Left* lateral intraventricular hemorrhage	*Left* venous angioma of choroid plexus	Acute repetitive focal seizures, postictal aphasia, homonymous hemianopsia	Poorly controlled focal epilepsy
4	Aguglia et al. (2008)	37 years	Female	Unilateral arm paresis, headaches	*Left* temporal hemorrhage	*Left* temporal angiomatous malformation	New-onset focal seizures, Todd’s paresis	Well-controlled focal epilepsy
5	Lopez et al. (2013)	20 months	male	Normal	Right subdural hemorrhage	Injury of the occipital region	Disturbance of consciousness	Free of seizures and stroke-like episodes
6	Nakajima et al. (2014)	2 years	Female	Epilepsy	*Right* thalamic hemorrhage	Obstruction of the superior sagittal sinus and *right* internal cerebral vein	Disturbance of consciousness, Hemiparesis	Rebleeding after 2 years
7	This case (2015)	62 years	male	Visual impairment in the right eye	*Left* thalamic hemorrhage	Obstruction of the superior sagittal sinus and *left* internal cerebral vein	New-onset headache, hemiparesis	Well improved hemiparesis

## Case presentation

**Fig. 1:**
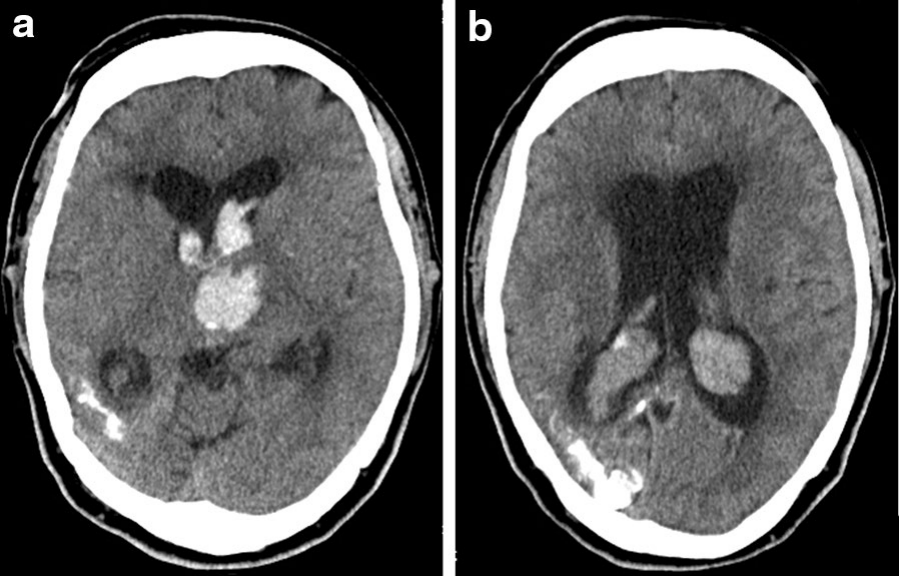
**a** CT on admission showing left thalamic hemorrhage and ventricular hemorrhage with acute hydrocephalus. **b** Bilateral choroid plexus enlargement and subcortical calcification were detected in the right temporo-occipital lobe

**Fig. 2:**
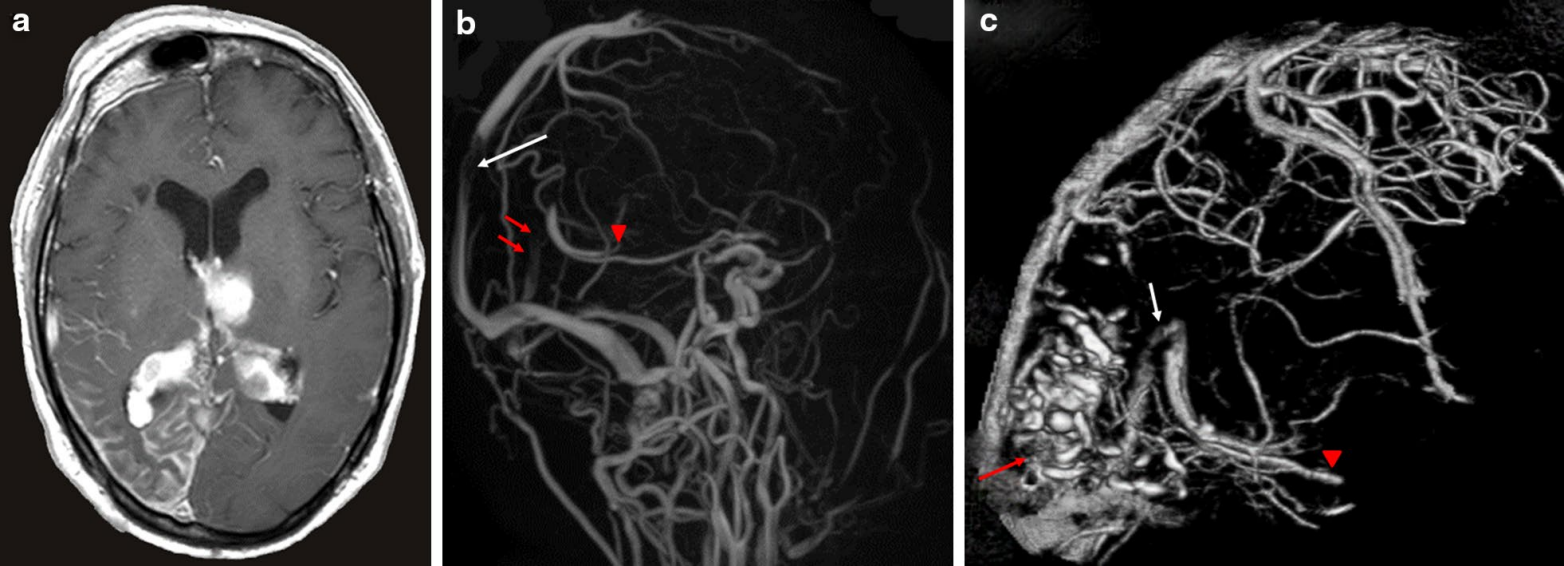
**a** Gadolinium-enhanced MRI showing left thalamic hemorrhage, leptomeningeal enhancement in the right temporo-occipital lobe, and choroid plexus thickening and enhancement on both sides. **b** Magnetic resonance venography (right to left view) showing steno-occlusion of the superior sagittal sinus (*white arrow*), straight sinus (*red arrows*), and left internal cerebral vein (*red arrow head*). **c** Three-dimensional CT angiography 2 weeks after the onset (right to left view) showing the patency of the straight sinus (*white arrow*) and left internal cerebral vein (*red arrow head*) and calcification in the right occipital lobe (*red arrow*)

A 62-year-old man suddenly fell into a coma, and was admitted to our hospital. At birth, he had a right-sided facial port-wine stain typical of SWS that involved the ophthalmic division of the trigeminal nerve. He had no history of ophthalmological or neurological clinical features including glaucoma, seizures, or stroke-like episodes, except for visual impairment in his right eye. His intraocular pressure was normal in both eyes. A fundus examination of both eyes showed no hemangiomas. He had not received any anticonvulsant or antiplatelet therapy, and had no history of heart disease or malignant tumors. On admission, his blood pressure was 191/96 mmHg and heart rate was 66 beats per minute in normal sinus rhythm. His body temperature was elevated to 37.1 °C and his body mass index was 25.6. His neurological status was assessed as GCS 6 (E1V1M4) with right-sided hemiparesis, and the National Institute of Health Stroke Scale score was 35 points. Laboratory findings suggested that he was dehydrated; hemoglobin 15.8 g/dl, hematocrit 46.3 %, blood urea nitrogen 31.7 mg/dl, and creatinine 1.70 mg/dl. Platelet-activating and coagulation factors were normal; platelet count 14.2 × 10^3^/μl, prothrombin time-international normalized ratio 0.94, and activated partial thromboplastin time 26.6 s. His serum D-dimer concentration was increased to 5.9 mg/dl. Computed tomography (CT) on admission showed left thalamic hemorrhage and ventricular hemorrhage with acute hydrocephalus (Fig. 1a). Bilateral choroid plexus enlargement and subcortical calcification in the right temporo-occipital lobe were also detected (Fig. 1b). Emergency ventricular drainage was performed. His disturbance of consciousness gradually improved to GCS 15 (E4V5M6) 7 days after the procedure, and right-side hemiparesis gradually subsided. We speculated that intracranial hemorrhage may have been derived from sinus thrombosis; therefore, magnetic resonance imaging (MRI) was performed. Gadolinium-enhanced MRI showed left thalamic hemorrhage, leptomeningeal enhancement in the right temporo-occipital lobe, and choroid plexus thickening with enhancement on both sides (Fig. 2a). Magnetic resonance venography showed steno-occlusion of the straight sinus, left internal cerebral vein (ICV), and superior sagittal sinus (SSS) (Fig. 2b). Rehydration therapy was performed to prevent sinus thrombosis. Two weeks after the onset, three-dimensional CT angiography revealed the patency of the straight sinus, left ICV, and SSS (Fig. 2c). His disturbance of consciousness gradually improved to GCS 15 (E4V5M6) 3 weeks after the procedure. Right-side hemiparesis gradually subsided, and he started to walk with the assistance of a side rail. The patient was referred to another hospital for further rehabilitation 28 days after the onset.

## Conclusions

SWS is a congenital neurocutaneous syndrome that is characterized by facial angioma (also known as a port wine stain) in the upper facial region and intracranial leptomeningeal angiomatosis is common. The Roach Scale has been used to classify encephalofacial angiomatosis, as follows: type I—facial and leptomeningeal angiomas (classic SWS), type II—facial angioma alone, and type III—isolated leptomeningeal-brain angioma. Our case was classified as type I, classic SWS.

Leptomeningeal angiomatosis results in a vascular steal that affects the subjacent cortex and white matter producing localized ischemia. Furthermore, deep venous occlusion of venous abnormalities in SWS is rare (Slasky et al. 2006).

 To the best of our knowledge, six cases of SWS with intracranial hemorrhage have been reported to date (Anderson and Duncan 1974; Pozzati et al. 1983; Dolkart and Bhat 1995; Aguglia et al. 2008; Lopez et al. 2013; Nakajima et al. 2014; Table 1). The median age of the patients was 15.1 years (range 1–62 years old). Intracranial hemorrhage occurs in most patients in their childhood, twenties, and thirties. Our case is the oldest among these cases. Regarding the premorbid neurological status, there have been three cases of SWS with epilepsy, one with unilateral arm paresis and headache, and one with visual impairment. The locations of hemorrhage were intraparenchymal in four cases, intraventricular in one, subarachnoid in one, and subdural in one.

The presumed etiologies of intracranial hemorrhage are described below.

Anderson et al. reported a 32-year-old woman with subarachnoid hemorrhage (SAH) (Anderson and Duncan 1974). There was rapid shunting through a capillary angiomatous malformation in the left basal ganglia and thalamic regions that was suspected of inducing the bleeding. The laterality of SAH was not observed. Pozzati et al. described a 9-year-old boy with intracerebral hemorrhage and ipsilateral ventricular penetration in the left parietal lobe (Pozzati et al. 1983). A giant aneurysm of the left posterior cerebral artery ruptured, and neck clipping was performed. Dolkart et al. reported a 24-year-old pregnant woman with left lateral intraventricular hemorrhage (Dolkart and Bhat 1995). Three days after delivery, intraventricular hemorrhage from left choroid plexus angioma developed suddenly. The hormonal or hemodynamic changes associated with pregnancy were suspected to be responsible for the bleeding. Aguglia et al. described a 37-year-old woman with intracranial hemorrhage in the left temporal lobe that was derived from the spontaneous thrombosis of left temporal angiomatous malformation (Aguglia et al. 2008). Lopez et al. reported a 20-month-old boy with right subdural hematoma that appeared to be associated with head trauma of an occipital lesion (Lopez et al. 2013). Nakajima et al. presented a 2-year-old girl with right thalamic hemorrhage (Nakajima et al. 2014). Obstruction of the superior sagittal sinus and right ICV were suspected to be the cause of bleeding.

The laterality of hemorrhage in most cases was derived from the ipsilateral side of the presumed etiology (Table 1). Angiomatous malformation, venous angioma of the choroid plexus, and sinus thrombosis are considered to be distinctive in SWS patients. However, the relationship between SWS and intracranial bleeding remains unknown in other cases.

Most of the signs and symptoms of SWS were headaches, new-onset focal seizure, and hemiparesis. SWS patients with intracranial hemorrhage mostly had good outcomes including being free of seizures and headaches. In our case, the sudden congestion of the straight sinus and left ICV may have caused left thalamic hemorrhage. Leptomeningeal angiomatosis was detected in the right temporo-occipital lobe, but was not considered to be the cause of breeding. Right-side hemiparesis gradually subsided and he started to walk with the assistance of a side rail.

Disease progression in SWS patients has been linked to recurrent thrombosis and resulting venous stasis. Cure et al. reported progressive venous occlusion in a neonate with SWS (Cure et al. 1995). Antiplatelet medications have been suggested to promote perfusion, and reduce thrombosis, which causes neurological injuries, in SWS patients (Garcia et al. 1981). We have to consider antiplatelet therapy in SWS patients prior to the development of sinus thrombosis.

In conclusion, we encountered a rare case of intracranial hemorrhage in a SWS patient. The outcomes of SWS patients with intracranial hemorrhage are not unfavorable. Antiplatelet medications have been suggested to promote perfusion and reduce thrombosis. Therefore, we need to consider the administration of antiplatelet medications to SWS patients prior to the development of sinus thrombosis.

## Abbreviations

SWS: Sturge–Weber syndrome
ICV: internal cerebral vein
CT: computed tomography
MRI: magnetic resonance imaging
SSS: superior sagittal sinus
SAH: subarachnoid hemorrhage
